# Correlates of Unwanted Births in Bangladesh: A Study through Path Analysis

**DOI:** 10.1371/journal.pone.0164007

**Published:** 2016-10-06

**Authors:** Tapan Kumar Roy, Brijesh P. Singh

**Affiliations:** 1 Department of Population Science and Human Resource Development, Rajshahi University, Rajshahi-6205, Bangladesh; 2 Faculty of Commerce, Banaras Hindu University, Varanasi-221005, Uttar Pradesh, India; London School of Economics and Political Science, UNITED KINGDOM

## Abstract

**Background:**

Unwanted birth is an important public health concern due to its negative association with adverse outcomes of mothers and children as well as socioeconomic development of a country. Although a number of studies have been investigated the determinants of unwanted births through logistic regression analysis, an extensive assessment using path model is lacking. In the current study, we applied path analysis to know the important covariates for unwanted births in Bangladesh.

**Methods:**

The study used data extracted from Bangladesh Demographic and Health Survey (BDHS) 2011. It considered sub-sample consisted of 7,972 women who had given most recent births five years preceding the date of interview or who were currently pregnant at survey time. Correlation analysis was used to find out the significant association with unwanted births. This study provided the factors affecting unwanted births in Bangladesh. The path model was used to determine the direct, indirect and total effects of socio-demographic factors on unwanted births.

**Results:**

The result exhibited that more than one-tenth of the recent births were unwanted in Bangladesh. The differentials of unwanted births were women’s age, education, age at marriage, religion, socioeconomic status, exposure of mass-media and use of family planning. In correlation analysis, it showed that unwanted births were positively correlated with women age and place of residence and these relationships were significant. On the contrary, unwanted births were inversely significantly correlated with education and social status. The total effects of endogenous variables such as women age, place of residence and use of family planning methods had favorable effect on unwanted births.

**Conclusion:**

Policymakers and program planners need to design programs and services carefully to reduce unwanted births in Bangladesh, especially, service should focus on helping those groups of women who were identified in the analysis as being at increased risks of unwanted births- older women, illiterate, low socioeconomic status, early age at marriage and rural poor susceptible women.

## Introduction

Unwanted birth is recognized as a birth when a woman doesn’t have any intention of becoming pregnant and deliver a birth. Also, unwanted births occurred when the women did not want to have any further births at all [1–3]. Unwanted birth occurs without the wish of woman, or after a woman has reached her desired family size and does not want any more children [4]. This birth occurs due to the fact that the growing desires to have smaller families, the unmet need for family planning, ineffectiveness of contraceptive methods, and unwanted sexual relations [5–9].

Unwanted birth occurs due to lack of awareness which may the result of unwanted pregnancy indirectly causes unsafe abortion which is one of the leading causes of maternal deaths, also this is one of the major reasons for induced abortion in adolescents, it accounts for about 13 percent of deaths worldwide and around three million teenage girls in undergo unsafe abortions every year [10]. It is the persistent health issue affecting the lives of several women and children across the globe. However, in Bangladesh, menstrual regulation (MR) is legal but the abortion service is not legally permitted. The quality of MR/abortion services in Bangladesh is reportedly poor and abortion-related complications contribute to about one-fourth of all maternal deaths in Bangladesh [5,11].

Unwanted birth is an important component of unplanned birth or unplanned pregnancy of reproductive health and such pregnancy has been used as a primary indicator of the state of reproductive health in Bangladesh [2,4,11]. This pregnancy is an important public health issue in both developing and developed countries in the world due to its negative association with the social and health outcomes for both mothers and children [1]. It is worthwhile to mention here that unwanted pregnancies are one of the important factor of maternal and child morbidity [8].

Bangladesh has achieved notable success in fertility decline, from a high level of 6.3 births per women in the mid-1970s to 2.3 births per women in 2011 but there has also been a sharp increase in the contraceptive users, from 7.7 percent to 61 percent during the same period [12]. However, in Bangladesh, the wanted fertility rate for Bangladesh is 1.6 which is quite lower than the total fertility rate 2.3 and 13 percent of pregnancies are unwanted [12]. This means that if all unwanted births could be eliminated, the total fertility rate would drop below the replacement level of fertility immediately [2]. In the current study, an attempt has been made to investigate the factors which are correlated to unwanted births and also explored the direct, indirect and total effects of socio-demographic factors on unwanted births in Bangladesh.

## Materials and Methods

The Bangladesh Demographic and Health Survey (BDHS) 2011 data has been used in this study and it covered nationally representative sample of 17,842 ever married women of age 15–49. For analysis purpose, a sub-sample of size 7,972 women has been considered. This study considers women whose most recent pregnancy occurred five years preceding the date of interview or women who were currently pregnant. The pregnant women were extracted using the question: “Are you pregnant now?” The answer was coded as either yes, no, or not sure. If she answered yes, she was considered as pregnant. Further, she was asked to respond the question, "At the time of becoming pregnant, did you want this birth then, did you want to wait until later, or did you not want to have any (more) children at all?" The women who wanted the birth then were considered under the wanted group, who desired birth, but later, were considered under the mistimed group, and who did not want to have any (more) children were considered under the unwanted group. There were 5,557 planned births, 1,327 were mistimed and 1,088 births were unwanted in this study.

To consider a model of causal (structural) relations for path analysis, some set of variables: women age (*X*_*1*_), place of residence (*X*_*2*_), religion (*X*_*3*_), socioeconomic status (*X*_*4*_), use of family planning methods (*X*_*5*_), women education (*X*_*6*_), age at first marriage (*X*_*7*_) and unwanted births (*X*_*8*_) have been selected from conception. Among the above selected variables, one variable may has a causal bearing on another, thus a correlation (or covariance) are observed in the data when all other relevant variables are controlled statistically. For this purpose, Pearson correlation analysis was used to identify significant relationships among the selected variables. Furthermore, path analysis was built on ordinary multiple regressions employed to know not only the effects of predictor (independent/explanatory) variable on dependent variable directly but also indirectly through one or more intervening variables. This technique also used to decompose the correlation coefficient, *r* into direct, indirect effects and total effects; to test the relative importance of each causal effect when compared to the others on the same dependent variable and to test the conceptual path model for their adequacy and the parsimony [13]. In this study, path analysis was used to examine the direct, indirect and total effects of the determinants of unwanted births.

In path analysis, the path coefficients are standardized regression coefficients in a system of linear regression equations, usually denoted *P*_*ij*_, where the first subscript represents the dependent variable and the second subscript indicates to the variable whose direct effect on the variable is measured. On the other hand, *P*_*ij*_ are path coefficients representing the direct effect of *j* on variable *i*. A path coefficient gives the proportion of the standard deviation of the dependent variable for which the independent variable is directly responsible [14]. In other words,

$$P_{ij}=\frac{\sigma_{j}}{\sigma_{i}}$$

where, *σ*_*j*_ and *σ*_*i*_ denote the standard deviation of the dependent and independent variables respectively. The path estimation equations are useful (i) in deriving path coefficients; (ii) in deriving the direct, indirect and residual and (iii) in predicting the implied correlation.

For analysis purposes, on the basis of the causal ordering of variables the selected set of variables are to divide into two groups- the exogenous variables {*X*_1_,*X*_2_,*X*_3_,*X*_4_,*X*_5_} and endogenous (dependent) variables {*X*_6_,*X*_7_,*X*_8_}. By using the above set of variables, it is important to construct a path diagram which visually expresses the hypothetical causal relationship between unwanted births and some selected socio-demographic variables. It presents the existence of causal framework interlinking the above selected variables with unwanted births. It is an extension of the multiple regression models and helps in understanding the effect of one variable on the other when they are arranged in a causal fashion. It also helps in understanding the important links between selected variables in the causal model. In this study, the path model is a recursive path model where each variable is assumed to be dependent upon all prior causal variables [13]. The following three regression models have been considered and the system of linear equations for the path model can be written as:

$$X_{6}=P_{65}X_{5}+P_{64}X_{4}+P_{63}X_{3}+P_{62}X_{2}+P_{61}X_{1}+P_{6u}R_{u}$$ (1)

$$X_{7}=P_{76}X_{6}+P_{75}X_{5}+P_{74}X_{4}+P_{73}X_{3}+P_{72}X_{2}+P_{71}X_{1}+P_{7v}R_{v}$$ (2)

$$X_{8}=P_{87}X_{7}+P_{86}X_{6}+P_{85}X_{5}+P_{84}X_{4}+P_{83}X_{3}+P_{82}X_{2}+P_{81}X_{1}+P_{8w}R_{w}$$ (3)

where, *P*_*ij*_ (*i* = 6, 7, 8; *j* = 1, 2, 3, 4, 5, 6, 7) are the path coefficients (regression parameters) and *R*_*u*_, *R*_*v*_ and *R*_*w*_ are the random error terms. The random error terms are mutually independent and are independent from their corresponding explanatory variables. In this study, the residual for path coefficients are derived from the regression equation as square root of (1−*R*^2^), where *R*^2^ is unadjusted and is known as multiple correlation coefficients of the regression equation for the endogenous variable [15]. According to Hu and Bentler (1999), the joint criteria for goodness of fit such as Comparative fit index (CFI) and the Root mean square error of approximation (RAMSEA) or (the Standardized root mean square residual (SRMR)) together help to converge on a decision for accepting a model have been considered [16]. The model fit indices values of CFI and RAMSEA (or SRMR) are reported 0.987 and 0.044 (0.015) respectively. Here, the model is over-identified, parameter estimates are obtained through generalized least squares estimation methods which assume multivariate normality and are asymptotically equivalent as well as asymptotically distribution-free methods. Statistical analyses were conducted using available SPSS program (21.0 for Windows, SPSS, Chicago, USA).

## Results

Table 1 shows percent distribution of unwanted births among ever married women whose most recent pregnancy occurred five years preceding the survey or who have been currently pregnant by some selected socio-demographic covariates. The result indicates that unwanted births are about 9.1 times higher among older women aged 25 years and more than their younger counterparts. Unwanted births among women decrease as education increases. Women with no education (28.1 percent) or who had primary education (17.6 percent) are more likely to have had an unwanted pregnancy than women with secondary or higher education (6.6 percent). Unwanted birth is higher among women who are married before age 18 years than those who have got marriage at age 18 and above years (15.1 percent vs. 9.2 percent).With the increases of age at marriage for unwanted birth among women, the percentage turned down. Unwanted births are higher among Muslim (14.4%) women as compared to their non-Muslim (7.0%) women. Unwanted births are varying with urban-rural residence. In urban area, about 11.5 percent women had unwanted births where as 14.6 percent women had unwanted births in rural area. Women with low economic status (18.3 percent) have more likely to have unwanted births than women with high socioeconomic status (9.7 percent). Unwanted pregnancies are more common among women who are using traditional methods and modern methods than women who are not using any family planning methods.

**Table 1:** Percent distribution of unwanted pregnancies/births of women aged 15–49 in last five years according to the selected socio-demographic characteristics, BDHS 2011.

Characteristics	Total (N)	Unwanted births (%)
***Women’s age***		
**<25**	4020	108 (2.7)
≥25	3952	980 (24.8)
***Women’s education***		
No education	1399	393 (28.1)
Primary	2369	416 (17.6)
Secondary	4204	279 (6.6)
***Age at first marriage***		
<18	5994	906 (15.1)
≥18	1978	182 (9.2)
***Religion***		
Muslim	7185	1033 (14.4)
Non-Muslim	787	55 (7.7)
***Place of residence***		
Urban	2550	294 (11.5)
Rural	5422	794 (14.6)
***Socioeconomic status***		
Low	3172	579 (18.3)
Medium	1523	192 (12.6)
High	3277	317 (9.7)
***Use of family planning methods***		
Not used	3099	371 (12.0)
Only traditional	501	99 (19.8)
Only modern	4372	618 (14.1)
***Pregnancy intention status***		
Planned	5557	69.7%
Mistimed	1327	16.6%
Unwanted	1088	13.7%

Pearson correlation coefficients (*r*) are known as total associations in path analysis which are used to examine the direction, strength and significance of linear relationships between variables [17]. Table 2 presents the results of pearson correlation coefficients (r) between some selected endogenous and exogenous variables. This pearson correlation coefficients are treated as total associations among variables and is considered to know the direction, strength and significance of linear relationships between variables. The results show that, unwanted births are significantly positively correlated with women age (0.269), and place of residence (0.101). However, the unwanted births are also significantly inversely correlated with socioeconomic status (-0.159), and women education (-0.355) at 1% level of significance. But, religion (-0.048), and age at marriage (-0.033) are inversely correlated with unwanted births though these show insignificant relationship. Use of family planning methods (0.024) is insignificantly positively correlated with unwanted births. It is also found that age at marriage is significantly positively correlated with women age (0.116), socioeconomic status (0.135), women education (0.136) and religion (0.076). On the other hand, age at marriage is significantly inversely correlated with place of residence (-0.088) at 1% level of significant. Age at marriage is insignificantly positively correlated with use of family planning methods (0.018). However, women education is significantly inversely correlated with women age (-0.131) and place of residence (-0.159). On the other hand, education is significantly positively correlated with socioeconomic status (0.412) at 1% level of significance. Education is insignificantly positively correlated with use of family planning methods (0.030) and inversely correlated with religion (-0.028). It shows that women age is significantly positively correlated with use of family planning methods (0.065) at 5% level of significance. However, use of family planning methods is insignificantly positively correlated with religion (0.048) and socioeconomic status (0.029). Though place of residence (-0.036) is insignificantly inversely correlated with use of family planning methods but socioeconomic status is significantly inversely correlated with place of residence (-0.449) at 1% level of significance. Women age (0.046) is positively correlated with socioeconomic status and religion (-0.032) but, there exists insignificant relationships.

**Table 2:** Results of correlation analysis among the selected variables with unwanted births.

Variables	X_1_	X_2_	X_3_	X_4_	X_5_	X_6_	X_7_	X_8_
***Women age (X***_***1***_***)***	1.000	0.013	0.020	0.046	0.065^b^	-0.131^a^	0.116^a^	0.269^a^
***Residence (X***_***2***_***)***		1.000	0.027	-0.449^a^	-0.036	-0.159^a^	-0.088^a^	0.101^a^
***Religion (X***_***3***_***)***			1.000	-0.032	0.048	-0.028	0.076^b^	-0.048
***Socioeconomic status (X***_***4***_***)***				1.000	0.029	0.412^a^	0.135^a^	-0.159^a^
***Use of FP methods (X***_***5***_***)***					1.000	0.030	0.018	0.024
***Women education (X***_***6***_***)***						1.000	0.136^a^	-0.355^a^
***Age at marriage (X***_***7***_***)***							1.000	-0.033
***Unwanted births (X***_***8***_***)***								1.000

Note:

^a^ Significant at 1% level and

^b^ Significant at 5% level.

The path coefficients are the direct effects of the determinant factors of unwanted births specified in regression Eqs (1), (2) and (3). This coefficient is defined as the ratio of the standard deviation of the effect due to a given cause to the total standard deviation of the effect. These coefficients (parameters) are estimated by using ordinary least-squares method. Thus, the equations for the fitted form of the illustrative path models to the data are given in the following:

$$X_{6}=0.030X_{5}+0.436X_{4}-0.013X_{3}+0.040X_{2}-0.154X_{1}$$ (4)

*P*-value (0.276) (0.000) (0.623) (0.190) (0.000)

$$R_{6.12345}^{2}=0.61$$

$$X_{7}=0.123X_{6}-0.002X_{5}+0.060X_{4}+0.081X_{3}-0.045X_{2}+0.128X_{1}$$ (5)

*P*-value (0.000) (0.960) (0.095) (0.007) (0.178) (0.000)

$$R_{7.123456}^{2}=0.49$$

$$X_{8}=-0.006X_{7}-0.309X_{6}+0.024X_{5}-0.026X_{4}-0.064X_{3}+0.039X_{2}+0.230X_{1}$$ (6)

*P*-value (0.822) (0.000) (0.385) (0.437) (0.020) (0.207) (0.000)

$$R_{8.1234567}^{2}=0.73$$

**Fig 1:**
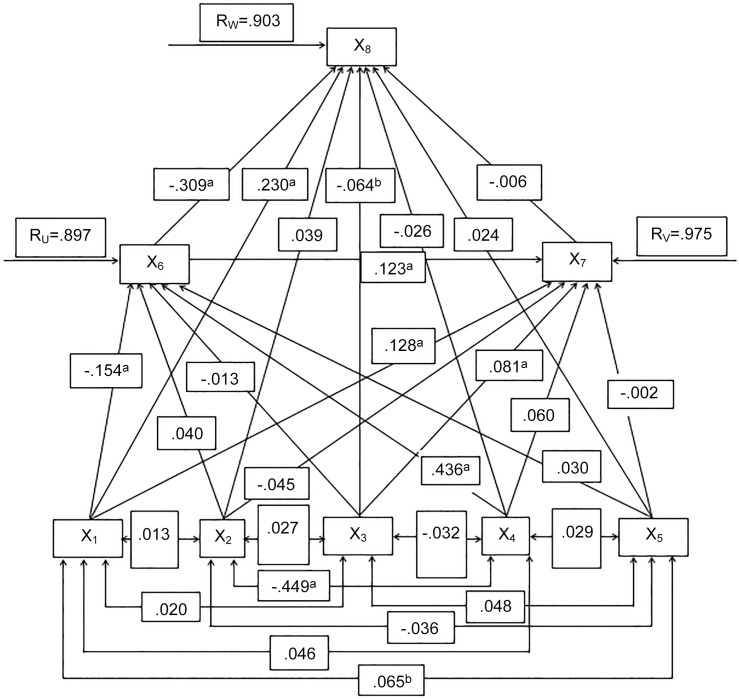
Path diagram for unwanted births and some selected socio-economic and demographic covariates in Bangladesh: Bangladesh Demographic and Health Survey, 2011.

The path analysis is based on path diagram where (i) the variables are arranged from bottom to top in such a way that all the endogenous variables are to the above of their exogenous variables; (ii) the unidirectional straight arrows called henceforth as causal paths that go from bottom to top represent the causal direct or net effects between exogenous and endogenous variables; (iii) on the other hand, the two-headed arrows represent the non-causal relationships among the exogenous variables and (iv) the un-measured, residual variables such as *R*_*u*_, *R*_*v*_ and *R*_*w*_ are also represented by unidirectional straight arrows coming from the residual variable to the endogenous variable. To employ path analysis, the variables involved in the path diagram are divided into three groups- the exogenous variables, endogenous variables and residual variables. The above categorized three types of variables are diagrammatically shown in Fig 1, which is the fitted form of the above path models. This path diagram shows interrelationship between unwanted births and various socio-demographic variables among ever married women in Bangladesh. The direct, indirect, and total effects and various path coefficients of the exogenous (independent) variables which are obtained from path analysis, and the interpretations of the effects of these factors on unwanted births are provided in Table 3.

**Table 3:** Effects of demographic and socio-economic variables on unwanted births through path analysis.

Endogenous Variable	Exogenous Variable	Total Effect	Non-Casual Effect	Indirect Effect Via		Direct Effect	Total Association
				X_6_	X_7_		
***X***_***6***_	***X***_***1***_	-0.154	-0.023	-		-0.154^a^	-0.131^a^
	***X***_***2***_	0.040	0.199	-		0.040	-0.159^a^
	***X***_***3***_	-0.013	0.015	-		-0.013	-0.028
	***X***_***4***_	0.436	0.024	-		0.436^a^	0.412^a^
	***X***_***5***_	0.030	0.000	-		0.030	0.030
***X***_***7***_	***X***_***1***_	0.109	-0.007	-0.019		0.128^a^	0.116^a^
	***X***_***2***_	-0.040	0.048	0.005		-0.045	-0.088^a^
	***X***_***3***_	0.079	0.003	-0.002		0.081^a^	0.076^b^
	***X***_***4***_	0.114	-0.021	0.054		0.060	0.135^a^
	***X***_***5***_	0.002	-0.016	0.004		-0.002	0.018
	***X***_***6***_	0.123	-0.013	-		0.123^a^	0.136^a^
***X***_***8***_	***X***_***1***_	0.280	0.011	0.048	0.0007	0.230^a^	0.269^a^
	***X***_***2***_	0.027	-0.074	-0.012	0.0003	0.039	0.101^a^
	***X***_***3***_	-0.061	-0.013	0.004	-0.0005	-0.064^b^	-0.048
	***X***_***4***_	-0.161	-0.002	-0.135	-0.0004	-0.026	-0.159^a^
	***X***_***5***_	0.015	-0.009	-0.009	0.0000	0.024	0.024
	***X***_***6***_	-0.310	0.045	-	-0.0007	-0.309^a^	-0.355^a^
	***X***_***7***_	-0.006	0.027	-	-	-0.006	-0.033

Note: (i) X_1_ = women age, X_2_ = place of residence,X_3_ = religion,X_4_ = socioeconomic status, X_5_ = use of family planning methods, X_6_ = women education, X_7_ = age at first marriage, X_8_ = unwanted births.

(ii) Non-Causal Effect = Total Effect—Total Association. Total effect = Direct effect + Indirect effect.

(iii) ^b^P < 0.05 and ^a^P < 0.01.

The total, non-causal, direct, and indirect effects and associations among endogenous and exogenous variables on the residual variable are found. In the path diagram, the total association with unwanted births is statistically significant with women age (*X*_*1*_), place of residence (*X*_*2*_), socioeconomic status (*X*_*4*_), and women education (*X*_*6*_). The women age (*X*_*1*_), place of residence (*X*_*2*_), religion (*X*_*3*_), socioeconomic status (*X*_*4*_), and women education (*X*_*6*_) are also statistically significant with age at marriage (*X*_*7*_) through total association. The total association with women education is statistically significant as women age (*X*_*1*_), place of residence (*X*_*2*_) and socioeconomic status (*X*_*4*_). In addition, the direct effects of women age (*X*_*1*_), religion (*X*_*3*_) and women education (*X*_*6*_) on unwanted births are statistically significant (Model 6). Moreover, the direct effects of women age (*X*_*1*_), religion (*X*_*3*_) and women education (*X*_*6*_) on age at marriage (*X*_*7*_) are statistically significant (Model 5) as are the direct effects of women age (*X*_*1*_) and socioeconomic status (*X*_*4*_) on women education (*X*_*6*_) [Model 4].

Women age and place of residence have favorable indirect effects and religion, socioeconomic status and women education have adverse indirect effects, on unwanted births through age at marriage. Religion and women age have favorable indirect effects, and place of residence, socioeconomic status and use of family planning methods have adverse indirect effects, on unwanted births through women education. Similarly, place of residence, socioeconomic status and use of family planning methods have favorable indirect effects. On the other hand, women age and religion have adverse effects on age at marriage through women education. Among determinants factors, women age, place of residence and use of family planning methods have favorable total effect on unwanted births, whereas, religion, socioeconomic status, women education and age at marriage have adverse total effects on unwanted births in Bangladesh.

Table 4 shows the percentage of indirect effect and direct effect on unwanted births through endogenous and exogenous variables. Women age on unwanted births about 17.22 percent is act through women education and about 0.25 percent is act through age at first marriage. Direct effect of women age on unwanted births is about 85.53 percent.

**Table 4:** Percentages of the total absolute effect on unwanted births through endogenous and exogenous variables.

Dependent Variable	Selected Variable	Percentage of Indirect Effect Via		Direct Effect
		X_6_	X_7_	
***X***_***8***_	***X***_***1***_	17.223	0.251	85.526
	***X***_***2***_	23.392	0.585	76.023
	***X***_***3***_	5.839	0.730	93.431
	***X***_***4***_	83.643	0.248	16.109
	***X***_***5***_	27.273	0.000	72.727
	***X***_***6***_	-----	0.226	99.774
	***X***_***7***_	-----	-----	100.00

Indirect effect of place of residence on unwanted births about 23.39 percent is act through women education and about 0.59 percent is act through age at first marriage. Place of residence on direct effect is about 76.02 percent with unwanted births. Religion about 5.84 percent is act through women education and about 0.73 percent is act through age at first marriage and direct effect is 93.43 percent. Indirect effect of socioeconomic status on unwanted births is 83.64 percent act through women education and 0.25 percent is act through age at first marriage. Direct effect of socioeconomic status is about 16.11 percent. The direct effect of use of family planning methods on unwanted births is about 72.73 percent and the indirect effect of 27.27 percent is act through women education. Women education has 99.77 percent direct effect on unwanted births and about 0.23 percent indirect effect is act through age at first marriage on unwanted births.

## Discussion and Conclusion

In this study, it has been observed that socio-demographic variables viz. women’s age, education, age at first marriage, religion, parity, residence, socioeconomic status, and use of family planning methods are important determinants of unwanted births in Bangladesh. The prevalence of unwanted births increased gradually with increasing women’s age in Bangladesh. The oldest women greater than equal to 25 years are more likely to report to have had unwanted births than the youngest women less than 25 years (24.8 percent vs. 2.7 percent). In a similar study, it shows that the likelihood of experiencing unwanted births among older women is higher than younger women due to majority of them already reached their desire number of children [18–19]. Another important socio-economic factors education plays great influence on unwanted births. Such unwanted births are higher among women with no education (28.1 percent) and those who had primary completed women (17.6 percent) respectively than women with secondary and higher education. Evidence from various studies it is found that that the unwanted births among the illiterate women are more common than educated mothers [20–22]. Demographic factor such as age at marriage has a significant impact on unwanted births in Bangladesh. If the age at marriage of women are raised it would have a negative effect on unwanted births. Unwanted births is advanced among women who are married before age 18 years than those who have got married at age 18 years and above (15.1 percent vs. 9.2 percent). The study indicates that women who have got married soon and their marriage duration is long that women have given more unwanted births which is the similar to the findings of other studies [19, 23–24]. Religion also has an important effect on unwanted births. From various studies on unintended pregnancies in Bangladesh, it is found that unwanted pregnancies are higher among Muslim women as compared to their non-Muslim colleagues [4,11,21]. Urban women have given more unwanted births than their rural counterparts which is also consistent with other studies conducted in Bangladesh [2, 19, 21, 24]. Socioeconomic status has an important contribution for unwanted births. Women have its low socioeconomic status (18.3 percent) have more likely to have unwanted births than women belong to high socioeconomic status (9.7 percent) which is similar with the findings shown in Bangladesh [2, 4, 11, 21] in Nepal [3] and in Ghana [22]. However, it is found that socio-economic variables are positively correlated with unwanted births. Unwanted births are more common among those women who had ever used any family planning methods than those women who don’t use any family planning methods. This is similar with the finding of several studies in Bangladesh [2, 4, 19, 21], in Ghana [22]. It shows highly significant relation with unwanted births and modern family planning methods use.

From the correlation analysis, it is found that unwanted births are significantly positively correlated with women age and place of residence. On the other hand, women education and socioeconomic status are significantly inversely correlated on unwanted births. The magnitude of these correlation coefficients is almost 0.269 for unwanted births on women age. The correlation coefficient is also significant with place of residence for unwanted births (0.101). The unwanted births are significantly negatively correlated with socioeconomic status (-0.159) and also women education (-0.355).

In this study, the causal link (direct, indirect and total effects) has been observed to examine among socio-economic and demographic variables through a prominent multivariate technique known as, path analysis. It has been found that eight paths for unwanted births out of each eighteen hypothetical paths are found to be statistically significant. The total effects of endogenous variables as women age, place of residence and use of family planning methods have favorable effect on unwanted births. The determinants factors of religion, socioeconomic status, women education and age at first marriage have adverse total effects on unwanted births. In addition, the direct effects of women age, religion and women education on unwanted births are statistically significant.

The main limitation of the study is absent of qualitative information from mothers regarding their feelings about unwanted births and biases inherent retrospective information on unwanted births. The data of cultural and psychological factors are also absent in this study. Unfortunately, Bangladesh Demographic and Health Survey data doesn’t collect above information. The finding also gives the need for further research to detect cultural and psychological factors associated women with higher risk of unwanted births. It may conclude that fertility impact especially on unwanted births is the main offender of unplanned births in Bangladesh. Policy planners should take appropriate policy in developing strategy to reduce unwanted births among married women of reproductive age in Bangladesh.
